# Supplementary material for: Neuronal parts list and wiring diagram for a visual system
Source: Nature. 2024 Oct 2;634(8032):166–80. doi: 10.1038/s41586-024-07981-1 (PMC11446827; doi:10.1038/s41586-024-07981-1)
Supplement: Supplementary file 5 — Cards for all cell types. Each card has 6 panels (clockwise): logical connectivity predicate diagram, numeric stats, illustration of a typical cell, followed by 3D rendering of all cells from three orthogonal viewpoints. Also available at Codex. [file 41586_2024_7981_MOESM5_ESM.zip › pdf/Am1.pdf]

*Am*1

predicate precision: 100%, recall: 100%

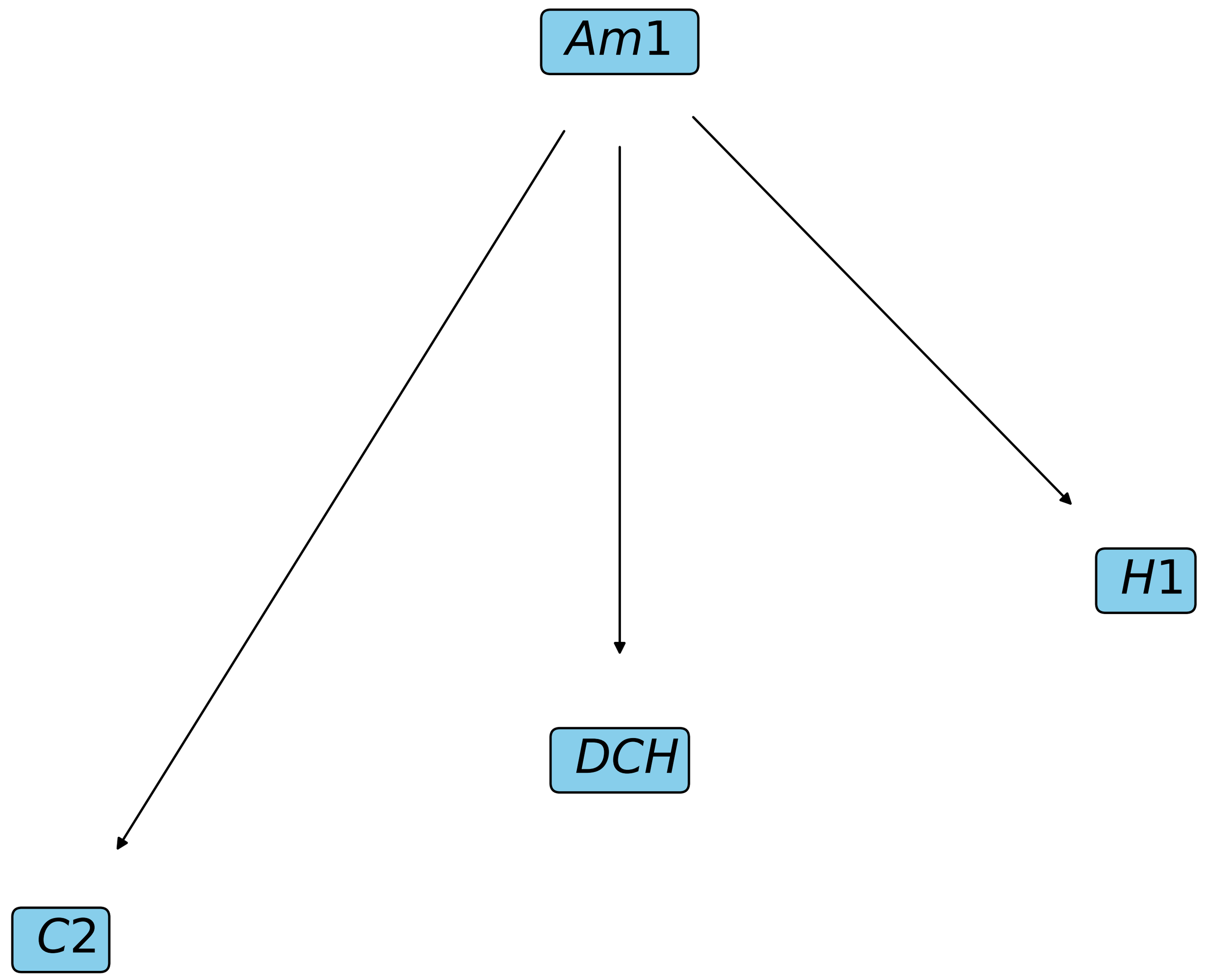

avg. stats

cells	1
NT prediction	GABA (100%)
top in/out regions	LOP (82%) / LOP (53%)
in/out degree	2,935 / 4,106
in/out synapses	34,915 / 26,408
cable len	$57,282\mu$
surface	$102,285\mu^2$
volume	$6,303\mu^3$

morphology

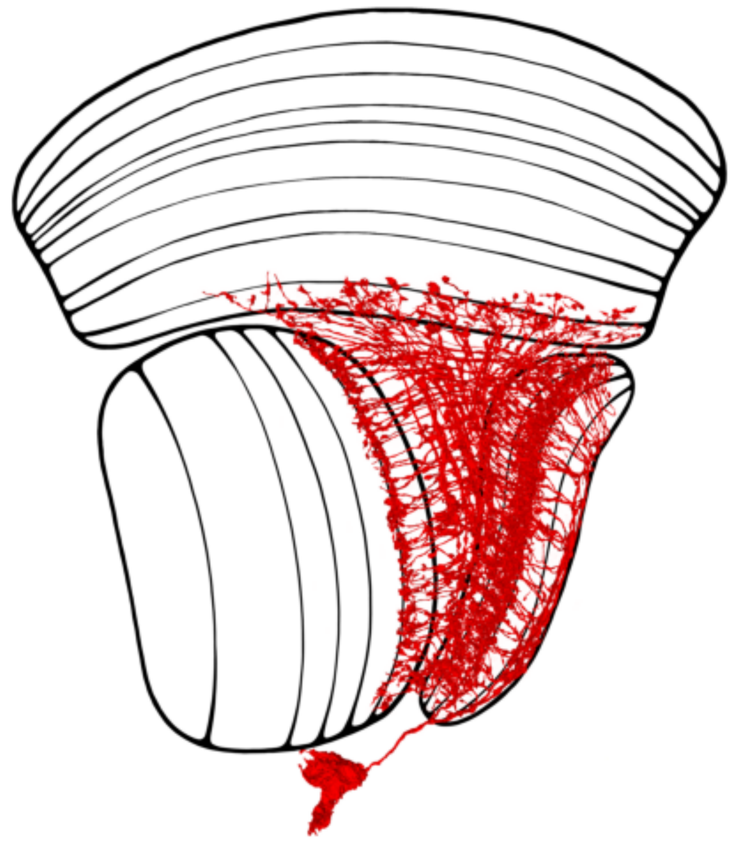

anterior view

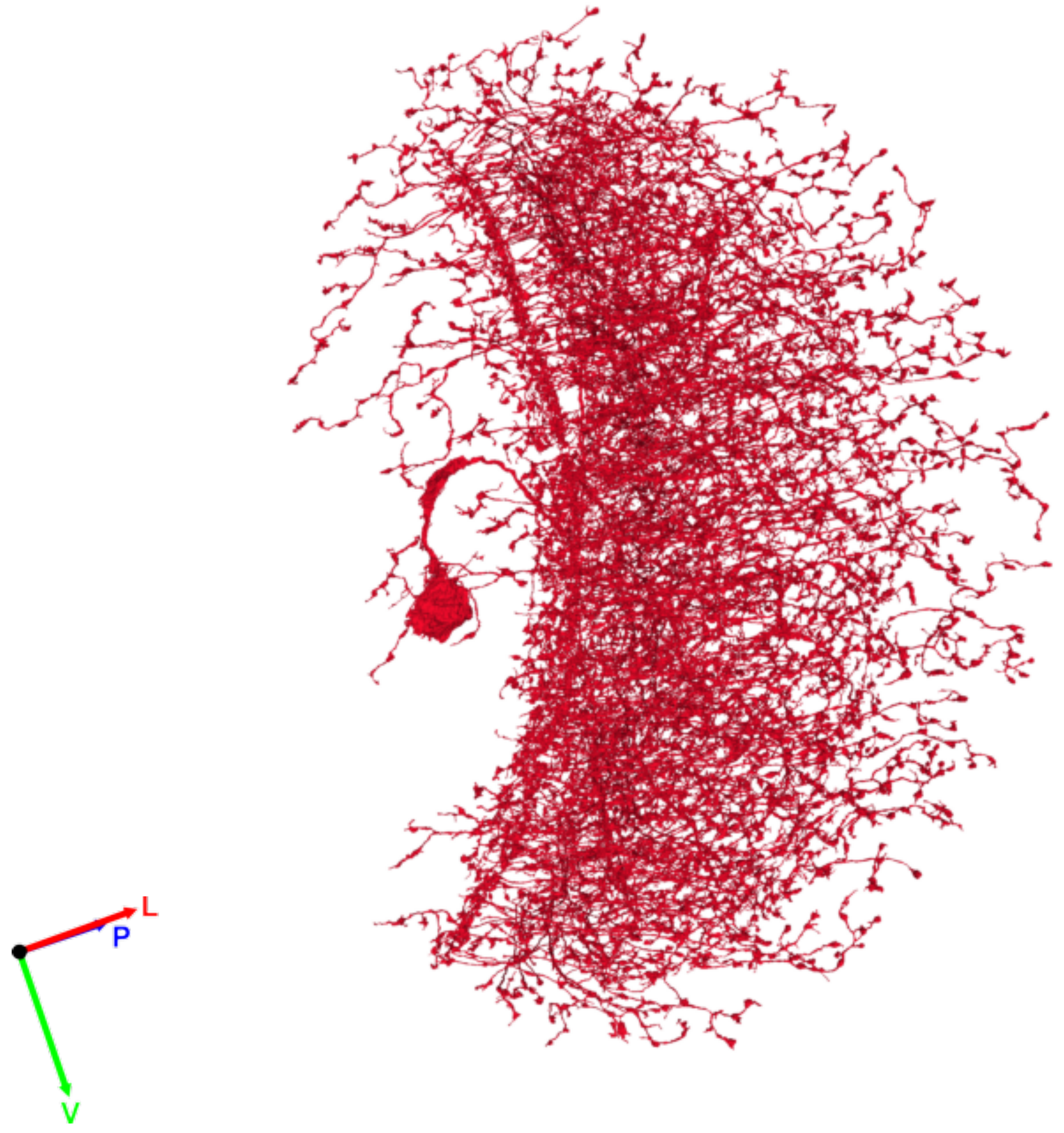

ventral view

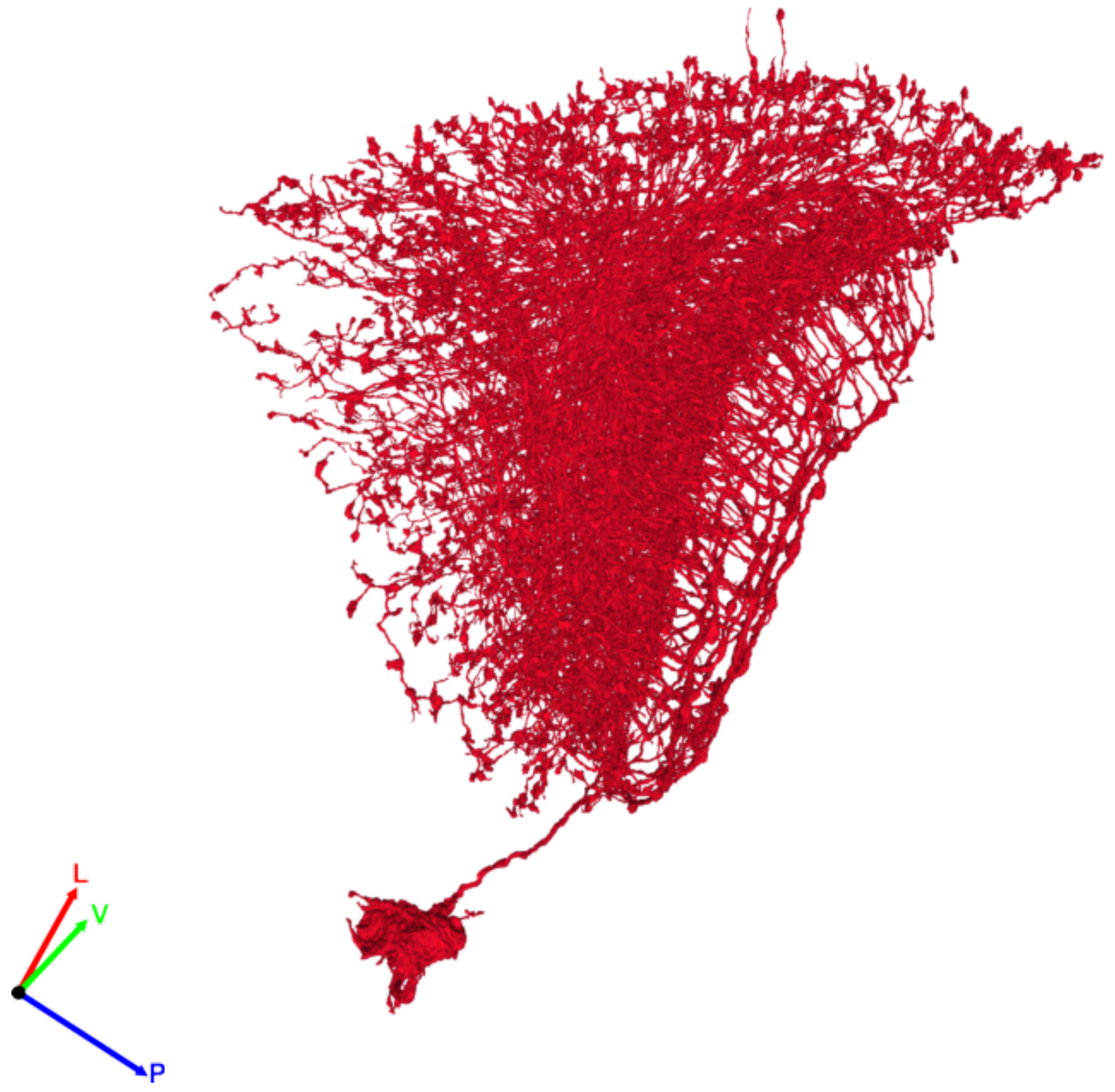

side view

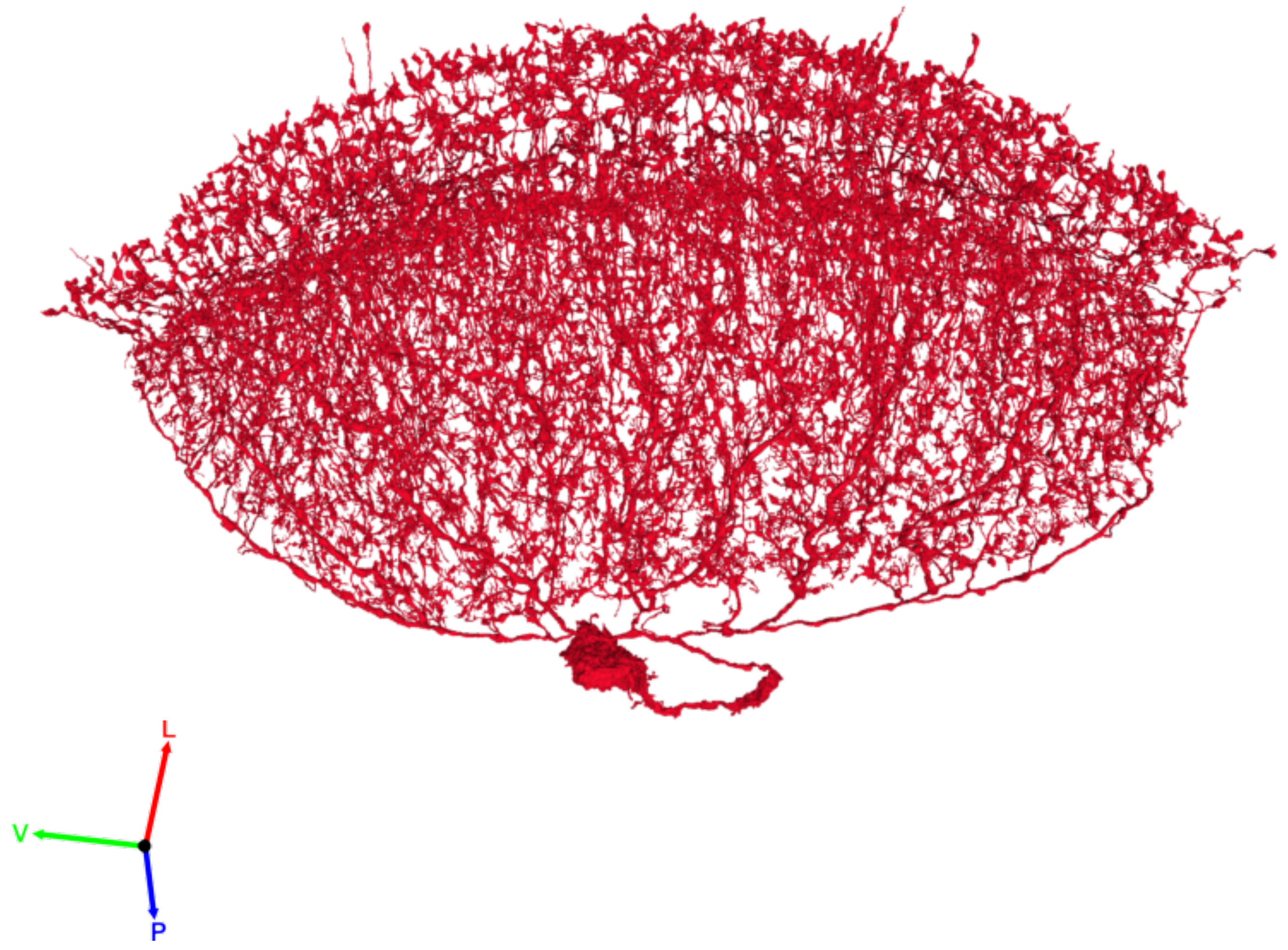

Medulla Lobula Lobula Plate Amacrine
